# Comorbidities, Social Impact, and Quality of Life in Tourette Syndrome

**DOI:** 10.3389/fpsyt.2016.00097

**Published:** 2016-06-06

**Authors:** Valsamma Eapen, Andrea E. Cavanna, Mary M. Robertson

**Affiliations:** ^1^School of Psychiatry, University of New South Wales, Sydney, NSW, Australia; ^2^Academic Unit of Child Psychiatry, Ingham Institute, Sydney South Western Local Health District, Sydney, NSW, Australia; ^3^Department of Neuropsychiatry, Birmingham and Solihull Mental Health NHS Foundation Trust, Birmingham, UK; ^4^Aston Brain Centre, School of Life and Health Sciences, Aston University, Birmingham, UK; ^5^Sobell Department of Motor Neuroscience and Movement Disorders, Institute of Neurology, University College London (UCL), London, UK; ^6^Neuropsychiatry, University College London (UCL), London, UK; ^7^St Georges Hospital and Medical School, London, UK; ^8^Department of Psychiatry, University of Cape Town, Cape Town, South Africa

**Keywords:** Tourette syndrome, attention-deficit hyperactivity disorder, obsessive compulsive disorder, autism spectrum disorder, comorbidity, psychopathology, psychosocial, quality of life

## Abstract

Tourette syndrome (TS) is more than having motor and vocal tics, and this review will examine the varied comorbidities as well as the social impact and quality of life (QoL) in individuals with TS. The relationship between any individual and his/her environment is complex, and this is further exaggerated in the case of a person with TS. For example, tics may play a significant role in shaping the person’s experiences, perceptions, and interactions with the environment. Furthermore, associated clinical features, comorbidities, and coexisting psychopathologies may compound or alter this relationship. In this regard, the common comorbidities include attention-deficit hyperactivity disorder and disruptive behaviors, obsessive compulsive disorder, and autism spectrum disorder, and coexistent problems include anxiety, depression, and low self-esteem, which can all lead to poorer psychosocial functioning and QoL. Thus, the symptoms of TS and the associated comorbid conditions may interact to result in a vicious cycle or a downward spiraling of negative experiences and poor QoL. The stigma and social maladjustment in TS and the social exclusion, bullying, and discrimination are considered to be caused in large part by misperceptions of the disorder by teachers, peers, and the wider community. Improved community and professional awareness about TS and related comorbidities and other psychopathologies as well as the provision of multidisciplinary services to meet the complex needs of this clinical population are critical. Future research to inform the risk and resilience factors for successful long-term outcomes is also warranted.

## Introduction

There are significant social and emotional sequelae to living with Tourette syndrome (TS), which can adversely affect the quality of life (QoL). Although majority of TS patients with mild forms of the disorder adapt to their symptoms and lead fulfilling lives, those with severe and persistent symptoms may experience significant negative impact on overall health and well-being. For example, an individual with TS may suffer from physical consequences such as the pain and discomfort of the repetitive movements and the stigma of the severe, violent, or socially inappropriate movements, vocalizations, or actions. Furthermore, they may become anxious particularly thinking about having the tics in front of others or become depressed from difficulties at school or lack of educational/vocational or employment opportunities. Lack of response to treatment or medication side effects as well as comorbidities may also add unique challenges.

## The Impact of Comorbidities and Coexistent Psychopathologies

The common comorbidities in TS include attention-deficit hyperactivity disorder (ADHD), obsessive compulsive disorder or behaviors (OCD/B), and autism spectrum disorder (ASD), whereas some of the common coexistent problems include anxiety, depression, substance abuse, childhood conduct disorder, and adult personality disorder (1). All of these can lower self-esteem directly or have consequences that may lead to poorer psychosocial functioning and QoL. For example, tics and comorbid ADHD may interact to create a vicious cycle of distractibility and inability to focus, due to both the efforts in trying to control the tics and due to inattention of ADHD; similarly, tics and comorbid severe obsessive compulsive disorder (OCD) may render an individual to check repeatedly, striving for perfection, and thus unable to finish school or office work (2). This may limit academic progress, while at the same time, negatively affecting the social outcomes and opportunities due to lack of education and under or unemployment. This may in turn create a downward spiraling of events compounded by poor frustration tolerance, impulsivity, and rage with consequent social exclusion, poor interpersonal, and family relationships. All of these can also precipitate or maintain comorbid mental health problems, drug and alcohol abuse, and even forensic encounters. Furthermore, the individual’s and their families’ QoL may be affected due to blame from delayed diagnosis or guilt from genetic etiology or wrong attributions about “parenting” or their own “TS” related behaviors including ADHD and obsessive compulsive features (clinical or subclinical) impacting on their ability to “parent” or “care” for the individual with TS.

One way of understanding the personal and social experiences of individuals with TS comes from the stories of people who have lived with TS for many years such as Joseph Bliss (3), and the writings of professionals with TS such as the neuroscientist Peter Hollenbeck (4), and physicians Lance Turtle (5), and Sam Zinner (6). Despite having marked symptoms, Bliss (3) received his diagnosis only at the age of 67 years. While identification and diagnosis of TS have improved in the last four decades since Bliss’s experience, clinicians working in the field continue to hear such stories about delay in diagnosis or misdiagnosis compounded by the lack of information, knowledge, and awareness about TS in the community, including among health professionals. Although tics remain the core feature of TS in the diagnostic classificatory systems (7), the presence of tics in the absence of other associated features and comorbidities occurs in only around 13% of cases (“pure-TS”) while the remaining (i.e., around 87%) have a number of associated features and comorbid disorders (“TS-plus”) (8). Comorbidities in TS can adversely affect the overall outcome and QoL in TS, and hence early recognition and appropriate management of the associated comorbidities and coexistent psychopathologies are critical.

## The Social Impact of TS on an Individual’s and Family’s Life

About one-third of TS patients have been reported to have social problems particularly due to potentially socially disabling features of TS, such as coprophenomena and also non-obscene socially inappropriate behaviors (NOSI), which is usually directed at a family member or familiar person at home or in a familiar setting (9). Other patients have self-injurious behaviors (10, 11) which can be difficult to treat and may compound social difficulties. These difficulties, plus shame, and embarrassment can also lead to difficulties outside the home or familiar settings and can have a negative impact on friendships and interpersonal relationships. For example, young people with TS have been found to have poorer peer relationships compared with their classmates and those with diabetes mellitus (12). Furthermore, in a clinical cohort of 16- to 54-year-old TS patients, problems with family relationships were reported in 29%, difficulties in making friends in 27%, social life in 20%, and being self-conscious in 15% (13). It has been found that parents of those with TS and comorbid behavioral disorder experience a greater impact on the family than those with uncomplicated TS (14). In this regard, increased Care Giver Burden and psychopathology have been reported in parents of young people with TS as compared with those with asthma (15). In addition, parents of young children with TS have been found to be significantly more likely to fall into the “parenting aggravation index” (e.g., feeling that their child is more difficult to care for than other children their age, feeling bothered by their child, and feeling angry with their child) category compared with those without TS (16). Moreover, another study observed that aggression and delinquency in the context of TS added unique contributions to impairment in social and family functioning, controlling for age, gender, and diagnostic status (17).

School problems have been noted in a number of clinic and community studies (18–20), and these have stressed the importance of teacher understanding and flexibility, as well as parent/school communication. It has also been found that the parents of children with TS considered tics to be the main cause of social maladaptation (21); finally, school-based intervention to improve knowledge and attitudes about TS has been found to enable prosocial behaviors in classmates while helping children with TS to embrace their condition (22).

A recent study comparing parental reports of TS youngsters with that of peers without TS found significantly higher rates of insecure peer attachment, problems in peer relationships, difficulty making friends, stigmatization, and lower levels of social functioning in the TS group, in particular, higher rates of the personality dimension “Neuroticism” acted as a significant barrier to friendship for individuals with TS (23). It has also been observed that parental perception of both tic frequency and intensity predicted tic-related functional impairment in several areas including family and peer relationships, school interference, and social endeavors with tic intensity predicting more variance across more domains than tic frequency (24). Another study (25) found that over half of the parents of TS patients reported one significant problem area due to the presence of tics, whereas over one-third reported two or more problem areas. The rate of non-tic-related impairment was very high, with 70% of parents reporting at least one problem area in the domains of school, home, or social activities (e.g., concentrating on school work, being prepared for class, taking tests or exams, or writing in class, doing household chores, sleeping at night, making new friends, and being with a group of strangers). In a UK study (26), significantly worse QoL was reported in a TS cohort compared with that of children in a normative sample with four main themes; “TS can be distressing and disabling,” “struggling to fit into society’s expectations of normal behavior,” “needing to control tics,” and “TS is one part of who I am.” Furthermore, high peer victimization and bullying have been reported in TS patients (27) as well as discrimination due to tics (28). Yet, another study (29) identified several main themes of social impact: these included more adverse experiences with TS than positive ones, pervasive misconceptions about TS symptoms, a desire for more understanding of TS by the public and understanding and supportive families, experiencing increased stress, academic challenges requiring accommodations, the active suppression of tics in school and in pubic, and finally, more complex social interactions with peers. It has also been observed that while young people felt the presence of their TS constantly, they often learnt to cope with their symptoms and other people’s reactions to them (30). Although they encountered problems when interacting with the wider peer network and expressed concerns around meeting new people and future employment, most of them had developed supportive friendships. The adolescents also described specific ways in which TS affects QoL and social interactions, and the effort it can take to cope successfully. Furthermore, low self-esteem has been linked with decreased QoL in all areas except for academic functionality (31). Considerable difficulties in socialization in TS patients have been reported, and it has been pointed out that the therapeutic elements must be identified by a change not only in environment but only in a child’s adaptation ability (32). In the light of these observations, it appears that treating both tic and non-tic-related impairments concurrently may improve functioning more so than treating the tic symptoms in isolation. In this regard, it has been noted (33) that treatment success should not only be assessed with the classic “tic-scales” but also with the global assessment of functioning (GAF) and TS-specific QoL scales. It is also important to control clinical symptoms and improve family environment to achieve better outcomes (34).

Moreover, Tourette syndrome patients have been found to exhibit insecure attachment with significantly higher scores in relationship anxiety and relationship avoidance and significantly higher aggression scores (35). A recent study of parents of young people with TS found that youth with TS are at increased risk for insecure peer attachment, which, in turn, might adversely affect the QoL outcomes (36). This study also observed that accurate identification of comorbidities is critical along with multidisciplinary support, as half of the parents of young people with TS had experienced stigmatization due to poor understanding about TS in the community including among those in the educational and health services (23, 37–39).

## Quality of Life in Tourette Syndrome

The wide-ranging impact of TS on health-related QoL of patients of all ages has been investigated in a number of dedicated studies since the turn of the millennium (13, 40) with the first study by Elstner et al. suggesting lower QoL in TS patients than in the general population (9). There is no consensus on the exact definition of QoL as it is affected by health; in addition, the relationship between clinical symptoms and QoL is neither simple nor direct (41). From an operational perspective, it has been proposed that subjective QoL can be conceptualized as the discrepancy between patients’ expectations about life and their actual experiences (42). Such a construct provides a useful framework for implementation in routine clinical practice; it is therefore not surprising that QoL is increasingly being used as a primary outcome measure for both health monitoring and active interventions for a range of medical conditions (43). Research has mainly focused on the burden of tic disorders and comorbid behavioral problems; the few controlled studies conducted to date have consistently shown that patients with TS have a poorer QoL than general population samples (13, 44, 45). Understandably, both the direct consequences of tic expression and the constant efforts related to their active suppression can be intrusive experiences affecting the individual’s well-being and their social interactions. Moreover, the high prevalence of comorbid behavioral problems in patients with TS is known to be associated with significant disease burden resulting in the subjective perception of poorer QoL (13).

The many clinical studies conducted in both children/adolescents (17, 24, 26, 28, 36, 39, 44–59) and admittedly fewer in adults (13, 60–66) with TS have, in general, shown similar results with lower QoL in TS. However, when examined in detail it becomes apparent that the different studies have yielded heterogeneous findings, especially with regard to the reciprocal contributions of tics and behavioral problems to specific domains of QoL. It is to be noted that the changes in arbitrary diagnostic criteria (e.g., DSM 111 > DSM-IV → DSM-IV-TR → DSM-5) over the long time period in which cited research has taken place may have contributed to the discrepancy in the findings, but taken together, the results of these studies suggest impairment across six general QoL themes as follows: physical, psychological, occupational, social, obsessional, and cognitive domains.

Severe tics have been reported to result in physical pain and in actual injuries. For example, findings from the Tourette Syndrome Impact Survey study, which involved both children and adults with TS, showed that the majority of respondents reported at least one tic that caused pain and indeed physical damage (64 and 60%, respectively), with significant correlations to reported tic severity (28, 65). Difficulties in carrying out activities of daily living, including self care, have also been documented among the consequences of problems in functional mobility and ability to perform exercises, especially as children mature to adolescence and adulthood (67). The presence of comorbid ADHD and OCD has been found to further affect the physical aspects of QoL, especially in children (44, 47), with few exceptions (48). Taken together, these findings suggest that the physical components of QoL should not be overlooked throughout the lifespan.

Psychological distress, feelings of frustration, and low mood in general are commonly experienced by patients with TS. The psychological domain of QoL has consistently been found to be significantly affected in the TS population compared with healthy controls. For example, 57% of adult patients with TS from a clinical sample reported problems with coexistent anxiety and depressive symptoms, with an odds ratio of 13 compared with age-matched controls (61). A study conducted in a clinical sample of children with TS showed that anxiety and depression were significantly more prevalent than in both healthy individuals (controls) and epilepsy control groups (44). It is thought that the increased prevalence of affective symptoms in TS, although not genetically linked, is probably multifactorial (68) rather than purely reactive to the psychosocial impairment and frustration caused by the chronic presence of tic symptoms (63). Psychological symptoms have been shown to be among the most important determinants of overall QoL (69), especially in adulthood (54, 60).

The negative impact of TS on QoL in children at school and in adults at work environment has also been investigated. The presence of comorbid conditions, particularly ADHD, has consistently been shown to affect school life (46, 47, 53). In addition, the spontaneous improvement or at least reduction of some of this comorbidity with age may contribute to explain the less pronounced impairment of QoL reported in adult working life (70, 71). For example, findings from the Tourette Syndrome Impact Survey study have shown that adults report milder interference with work productivity compared with the level of academic interference noted by the child population (28, 65). The development of coping strategies through adolescence has been found to improve satisfaction in the workplace (30), although dissatisfaction with school experiences can have far-reaching implications, possibly influencing future career or occupational choices or even employment status (72).

Relationships with family and friends are also important in life and indeed are also components of the social domain of QoL. Specifically, healthy family functioning has been recognized as integral to long-term social and emotional stability in children with TS (73). Multiple studies have shown that younger patients can often feel responsible for family arguments as a result of their TS symptoms and can therefore be more likely to avoid communication with their parents (44, 48, 57), possibly resulting in increased insecurity and exacerbated problems over time (57). In turn, one study conducted in adult patients with TS showed that 29% of participants had felt unsupported by their family about their condition (13). Of importance is that patients of all ages have reported a higher interference from TS within peer friendships than in family relationships (28, 65). The former way will result in potential difficulties in the formation of intimate or meaningful relationships which are an important part of adult life (63). However, the full extent of the social impact of the comorbidities of TS remains difficult to determine and quantify, especially in the case of adults with comorbid OCD (40, 74).

Nevertheless, the development of disease-specific QoL measures, such as the GTS-QoL in adults and the GTS-QoL-C&A in children, has enabled researchers to more sensitively assess the impact of repetitive behaviors and comorbid OCD on the overall perception of QoL in patients with TS (54, 60). Results from studies using disease-specific measures seem to indicate a decrease in the perceived impact of OCD on QoL as patients develop to adulthood, in the absence of decreased symptom severity, possibly suggesting the development of more effective coping strategies over time (51, 64).

Reduced concentration, forgetfulness, and inability to complete important tasks are important cognitive aspects of QoL. Although age-dependent improvement of comorbid ADHD seems likely to have a significant impact on cognitive functioning (71), results from the Tourette Syndrome Impact Survey study highlighted a significant correlation between tic severity and cognitive domain scores (65). Studies conducted using the GTS-QoL further suggested that QoL perception in adulthood is more deeply affected by cognitive factors than in children (54, 60). These findings suggest that complex interaction between tics and cognitive function in determining QoL across the lifespan deserves further investigation in future studies.

## Conclusion

The social impact of TS is varied, and there are a number of TS patients who are known to us and are reported in the literature who cope and adapt well, with many using creativity or humor to their advantage, or by focusing on something that they are good at or enjoy doing such as leisure activities, sports, or academic or artistic pursuits. However, in those with severe forms of the disorder and with severe comorbidities, TS may interfere with the individual’s everyday life and activities of school, home, or work, such as being educated to their full potential, obtaining a job/career, gaining independence, and having meaningful relationships with family and friends. There are a number of factors that contribute to outcomes in terms of social adjustment and QoL, although treatment for tics or better coping strategies may be positively correlated with functional improvement, particular attention to the complex interaction with comorbidities is critical to successful outcomes. For example, fidgetiness may be part of tics or due to ADHD or both; coprolalia and disruptive behaviors may well attract negative consequences such as disciplinary action in children or stigma and social embarrassment in adults (2). In this regard, supportive environments, anticipatory guidance, as well as appropriate emotional, behavioral, and learning supports are indicated to overcome the challenges confronting those with TS. Education of health and other professionals as well as implementation of community awareness programs are needed along with research to gain better understanding of the factors that contribute to better long-term outcomes. Thus, we suggest that future research should examine the possible influence of successful treatment on outcomes such as pharmacological intervention for symptom control or indeed improving a sense of personal mastery through skill building in comprehensive behavioral intervention for tics (CBIT); this must be conducted in tandem with research on the quality, duration, and effect of early supportive services on later QoL.

## Author Contributions

VE, AC, and MR jointly wrote the manuscript, each taking charge of a subsection.

## Conflict of Interest Statement

The authors declare that the research was conducted in the absence of any commercial or financial relationships that could be construed as a potential conflict of interest.
